# Supplementary material for: Assessment of Cardiopulmonary Bypass Duration Improves Novel Biomarker Detection for Predicting Postoperative Acute Kidney Injury after Cardiovascular Surgery
Source: J Clin Med. 2021 Jun 22;10(13):2741. doi: 10.3390/jcm10132741 (PMC8268369; doi:10.3390/jcm10132741)
Supplement: Supplementary file 1 [file jcm-10-02741-s001.zip › jcm-1240155-supplementary.pdf]

Supplementary Materials

Supplementary Table S1. Baseline characteristics of the patients, stratified by AKI after cardiovascular surgery

	All patient			ACEF≥1.1		
	AKI (n =59)	Non-AKI (n =85)	*p*	AKI (n =26)	Non-AKI (n =32)	*p*
Baseline characters						
Age, year	63.6±12.8	60.9±12.8	0.216 ‡	69.7±12.2	69.3±9.8	0.892‡
Gender, male	38(64.4%)	57(67.0%)	0.741	18(69.2%)	22(68.8%)	0.969
Underlying disease						
Diabetes mellitus	20(33.9%)	33(38.8%)	0.547	14(53.8%)	19(59.4%)	0.672
Pre-operation examination data						
Hemoglobin, g/dL	11.6 ± 2.4	13.1 ± 2.2	<0.001 ‡	11.5±2.0	12.6±1.8	0.037‡
Platelet, 1000/uL	223.1 ± 91.0	228.3 ± 75.2	0.715 ‡	217.5±89.3	223.6±80.5	0.789‡
White blood cell count, /uL	7650.0 ± 2968.5	7302.3 ± 2322.2	0.434 ‡	7042.3±2313.8	6887.1±2433.2	0.807‡
Creatinine, mg/dL	0.8 [0.7; 1.2]	0.8 [0.7; 1.0]	0.509 †	1.0 [0.8; 1.2]	0.8 [0.7; 1.1]	0.351†
eGFR, ml/min*	83.0 ± 24.0	88.4 ± 21.5	0.159 ‡	73.6±22.8	78.9±19.1	0.337‡
ALT, mg/dL	29.0 [18.0; 40.0]	24.5 [18.0; 35.2]	0.544 †	27.0 [17.3; 39.8]	20.0 [15.0; 30.0]	0.482†
LVEF, %	62 [47; 68]	65 [55; 72]	0.211 †	45 [40; 59]	52 [37; 60]	0.782†
ACEF score	1.1 [0.9; 1.3]	1.0 [0.8; 1.2]	0.091 †	1.4 [1.3; 1.9]	1.3 [1.1; 1.7]	0.233†
Surgical type						
Aortic surgery	1(1.7%)	0(0%)		0(0%)	0(0%)	
CABG	15(25.4%)	38(44.7%)		11(42.3%)	18(56.3%)	
CABG and valve surgery	6(10.2%)	3(3.5%)		4(15.4%)	1(3.1%)	
Valves surgery	36(61.0%)	44(51.8%)		11(42.3%)	13(40.6%)	
Others	1(1.7%)	0(0%)		0(0%)	0(0%)	
Surgical related factors						
CPB time, mins	165.8 ± 62.5	118.9 ± 46.3	<0.001 ‡	144.35±41.17	110.16±43.36	0.004‡
Clamp time, mins	98 [41; 143]	71 [0; 108]	0.003 †	83 [0; 113]	0 [0; 105]	0.100†
HTK perfusion	46 (78.0%)	52 (61.2%)	0.034	19(73.1%)	17(65.4%)	0.119
ICU stay, days	3.0 [2.0; 5.0]	1.0 [1.0; 2.0]	<0.001 †	3.5 [2.0; 5.0]	1.5 [1.0; 3.0]	<0.001†
Mortality	1(1.2%)	4(6.8%)	0.072	3(11.5%)	0(0%)	0.048

Data were presented as frequency (percentage), mean ± standard deviation or median [25th, 75th percentile].

*pre-operation eGFR was estimated by creatinine via CKD-EPI

‡ Student t-test; † Mann–Whitney u-test

ACEF score: age, creatinine, ejection fraction score; AKI: acute kidney injury; ALT: alanine aminotransferase; CABG: coronary artery bypass graft; CPB: cardiopulmonary bypass; eGFR: estimated Glomerular filtration rate; HTK: histidine-ketoglutarate-tryptophan; ICU: intensive care unit; LVEF: left ventricular ejection fraction

Supplementary Table S2. Baseline characteristics of the patients, stratified by ACEF score

	ACEF≥1.1 (*n* =60)	ACEF<1.1 (*n* =81)	*p*
Baseline characters			
Age, year	69.4±10.7	57.1±11.3	<0.001‡
Gender, male	42(70.0%)	51(63.0%)	0.383
Underlying disease			
Diabetes mellitus, n(%)	33(55.0%)	19(23.5%)	<0.001
Pre-operation data			
Hemoglobin, g/dL	12.2±2.0	12.7±2.6	0.133‡
Platelet, 1000/uL	220.5±83.9	230.2±81.6	0.495‡
White blood cell count, /uL	6906.8±2339.0	7822.5±2754.4	0.041‡
Creatinine, mg/dL	0.9 [0.7; 1.2]	0.8 [0.7; 0.9]	0.013†
eGFR, ml/min*	76.5±20.7	92.9±21.2	<0.001‡
ALT, mg/dL	21.0 [16.5; 33.0]	29.0 [18.8; 38.5]	0.080†
LVEF	49 [39; 60]	69 [65; 75]	<0.001†
ACEF score	1.3 [1.2; 1.8]	0.9 [0.7; 1.0]	<0.001†
Surgical type			
Aortic surgery, n(%)	0(0%)	1(1.2%)	
CABG, n(%)	30(50.0%)	22(27.2%)	
CABG and valve surgery, n(%)	5(8.3%)	4(4.9%)	
Valves surgery, n(%)	25(41.7%)	54(66.7%)	
Others , n(%)	0(0%)	0(0%)	
Surgical related factors			
CPB time, mins	124.6±45.0	148.2±64.4	0.017‡
Clamp time, mins	71 [0; 108]	94 [0; 122]	0.015†
HTK perfusion	37(61.7%)	60(74.1%)	0.116
Post-operation biomarker			
L-FABP time 1	61.7 [22.3; 124.2]	46.0 [21.4; 196.8]	0.828†
L-FABP time 2	96.78 [25.26; 199.1]	55.9 [17.4; 122.0]	0.241†
ICU stay, days	2.0 [1.0; 4.0]	2.0 [1.0; 3.0]	0.706†
AKI, n(%)	26(43.3%)	32(39.5%)	0.648
KDIGO stage 1	16(26.7%)	23(28.4%)	
KDIGO stage 2	6(10.0%)	3(3.7%)	
KDIGO stage 3	4(6.7%)	6(7.4%)	
Renal replacement therapy	3(5%)	0(0%)	0.042
Mortality	3(5%)	2(2.5%)	0.422

Data were presented as frequency (percentage), mean ± standard deviation or median [25th, 75th percentile].

*pre-operation eGFR was estimated by creatinine via CKD-EPI

‡ Student t-test; † Mann–Whitney u-test

ACEF score: age, creatinine, ejection fraction score; AKI: acute kidney injury; ALT: alanine aminotransferase; CABG: coronary artery bypass graft; CPB: cardiopulmonary bypass; eGFR: estimated Glomerular filtration rate; HTK: histidine-ketoglutarate-tryptophan; ICU: intensive care unit; KDIGO: kidney disease improving global outcomes; L-FABP: liver type fatty acid binding protein, LVEF: left ventricular ejection fraction

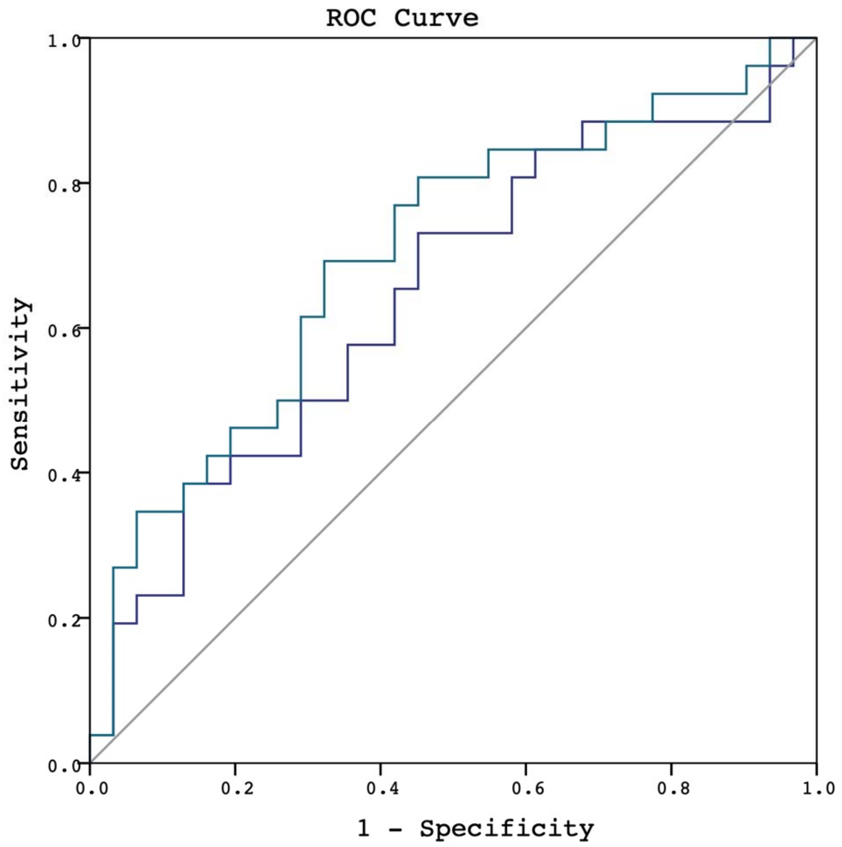

Supplementary Figure S1. AUROC for L-FABP in discriminating postoperative AKI in patients with ACEF score ≥1.1, the urinary L-FABP at the first timepoint is shown by the blue line and the L-FABP at the second timepoint is shown by the green line.
